# Functional Biomaterials Derived from Protein Liquid–Liquid Phase Separation and Liquid‐to‐Solid Transition

**DOI:** 10.1002/adma.202414703

**Published:** 2025-02-09

**Authors:** Tianchen Li, Dea Ilhamsyah, Benedict Tai, Yi Shen

**Affiliations:** ^1^ School of Chemical and Biomolecular Engineering The University of Sydney Darlington NSW 2008 Australia; ^2^ The University of Sydney Nano Institute The University of Sydney Sydney NSW 2006 Australia

**Keywords:** liquid–liquid phase separation, liquid‐to‐solid transition, protein condensate, protein self‐assembly

## Abstract

Protein phase transitions play a vital role in both cellular functions and pathogenesis. Dispersed proteins can undergo liquid–liquid phase separation to form condensates, a process that is reversible and highly regulated within cells. The formation and physicochemical properties of these condensates, such as composition, viscosity, and multiphase miscibility, are precisely modulated to fulfill specific biological functions. However, protein condensates can undergo a further liquid‐to‐solid state, forming β‐sheet‐rich aggregates that may disrupt cellular function and lead to diseases. While this phenomenon is crucial for biological processes and has significant implications for neurodegenerative diseases, the phase behavior of naturally derived or engineered proteins and polypeptides also presents opportunities for developing high‐performance, multifunctional materials at various scales. Additionally, the unique molecular recruitment capabilities of condensates inspire innovative advancements in biomaterial design for applications in drug discovery, delivery, and biosynthesis. This work highlights recent progress in understanding the mechanisms underlying protein phase behavior, particularly how it responds to internal molecular changes and external physical stimuli. Furthermore, the fabrication of multifunctional materials derived from diverse protein sources through controlled phase transitions is demonstrated.

## Graphical abstract

Protein can undergo liquid–liquid phase separation and liquid‐to‐solid transition to form liquid condensates and solid aggregates. These phase transitions can be influenced by post‐translational modifications, mutations, and various environmental factors. Effective modulation of protein phase behavior offers promising applications in drug discovery, delivery, and fabrication of multifunctional protein‐based liquid and solid materials.

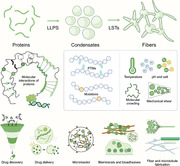

## 1 Introduction

**Figure 1:**
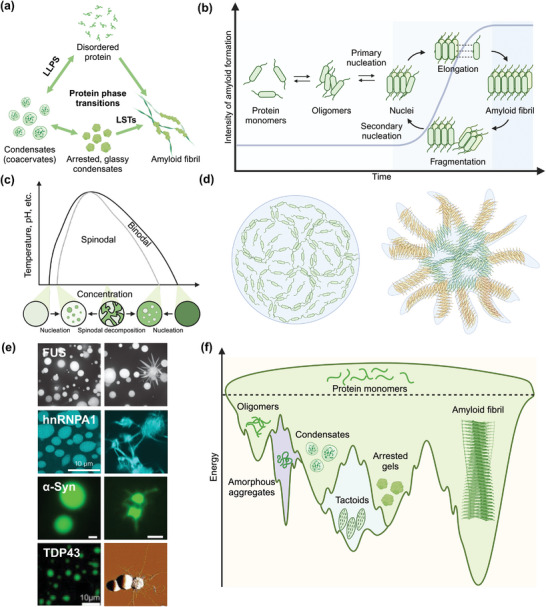
a) Overall protein phase transitions. b) A schematic view of the amyloid fibril formation process via classical nucleation theory. c) Phase diagram of protein LLPS. Reproduced with permission.^[^ ^21^ ^]^ Copyright 2018, Elsevier. d) Schematic structure of gel‐like (left) and amyloid fibril‐rich structure after LSTs of condensates (right). The protein molecules labeled in green indicate the region inside the condensates, and the ones in brown show the amyloid fibrils expanded outward the solidified condensates. e) The normal (left) and aberrant (right) condensate of FUS, hnRNPA1, α‐synuclein, and TDP‐43. The images of FUS. Reproduced with permission.^[^ ^11^ ^]^ Copyright 2015, Elsevier. The images of hnRNPA1. Reproduced under the terms of the CC‐BY 4.0 license.^[^ ^12^ ^]^ Copyright 2023, Springer Nature. The images of α‐synuclein. Reproduced under the terms of the CC‐BY 4.0 license.^[^ ^13^ ^]^ Copyright 2023, AAAS. The images of TDP‐43. Reproduced under the terms of the CC‐BY license.^[^ ^14^ ^]^ Copyright 2019, Elsevier. f) The energy landscape diagram of protein phase transition products.

Proteins are biomolecules with complex structures that can undergo phase transitions like water molecules (**Figure** **1**a). Amyloid fibril is one of the most studied protein solid states due to its relevance in neurodegenerative disorders. The self‐assembled cross β‐sheet aggregates have high thermal stability with minimum reversibility, making the associated diseases difficult to treat. Interestingly, amyloid‐like structures can also be found in natural materials, such as silk fibers^[^ ^1^ ^]^ and mussel byssus thread.^[^ ^2^ ^]^ The β‐sheet rich nature equipped these materials with extradentary mechanical and optical properties. In fact, a broad range of proteins can be engineered into nanofibrils, such as proteins from soybeans,^[^ ^3^ ^]^ bovine milk,^[^ ^4^ ^]^ and egg whites.^[^ ^5^ ^]^ Many of them have been used as building blocks for the development of innovative biomaterials with excellent mechanical strength, as described in previous studies.^[^ ^6^ ^]^

Protein condensates, as the liquid state of protein molecules, have recently been discovered to be involved in many essential biological processes in cells. The intercellular biomolecules, including millions of protein molecules and other biopolymers, are not uniformly distributed in cells but organized into concentrated liquid droplets, named biomolecular condensates, via liquid–liquid phase separation (LLPS).^[^ ^7^, ^8^ ^]^ They can recruit or release proteins, peptides, nucleic acids, and other chemicals on demand. They have variant material properties and can be formed, dissolved, merged or separated from one another repeatedly for different purposes.^[^ ^9^ ^]^ However, the protein condensates are usually unstable and can transit into arrested states over time. This process is defined as liquid‐to‐solid transitions (LSTs), sometimes called “aging.” Changes in protein sequence due to mutation, defects of condensate regulators, and physiochemical condition fluctuation inside the cells can accelerate this transition, leading to irreversible aggregate formation.^[^ ^10^ ^]^ It is believed that protein condensates are likely to be one of the precursors during amyloid fibril formation as a result of LSTs. This is further supported by the discovery of LLPS of several neurodegenerative disease‐related proteins and their aging into amyloid fibrils.^[^ ^11^, ^12^, ^13^, ^14^ ^]^

Protein condensates have been used as a tool for drug discovery and delivery due to their unique liquid‐like property and specific recruitment of molecules. The dynamic nature of condensates allows them to penetrate cell membranes with a direct cytosolic pathway.^[^ ^15^ ^]^ The composition of the condensates can also alter their phase behavior. All these properties make them perfect candidates for drug carriers and screening tools for drug discovery. Liquid states of protein are also widely discovered outside the cells, such as the glands of silkworms, spiders, mussels, and many other species. Natural bioactivities, such as silk spinning and underwater adhesions of mussels, utilize the process of protein LLPS and LSTs. In particular, fiber‐forming protein is stored and concentrated in condensates. When needed, these liquid droplets can be secreted, undergo LSTs triggered by external stimuli, and self‐assemble into a series of micro‐ or macroscale structures, such as fibers and gels, for multiple purposes.^[^ ^16^, ^17^ ^]^ However, very limited materials are designed and engineered based on such phase behavior. As the understanding of protein phase transition develops, people have begun to utilize this behavior in treating protein‐aggregation‐related diseases and develop bioinspired materials for desired properties. In this review, we focus our discussion on the protein‐based materials derived from LLPS and LSTs. We investigate the fundamental basics behind LLPS and LSTs, linking the disease mechanism with biomaterial innovation. We overview recent examples, demonstrating the design of novel protein/peptide‐based materials based on protein phase behavior.

## 2 Fundamental Basics of Protein Phase Transition

### 2.1 Protein Aggregation via Nucleation

Due to its relevance to diseases, decades of efforts have been made to reveal the structure of amyloid fibrils and the kinetics of amyloid aggregation. It is now widely accepted that protein self‐assembly into amyloid fibrils fits the classical nucleation theory (Figure 1b).^[^ ^18^ ^]^ During the primary nucleation, dispersed protein molecules interact to form oligomers, which then slowly self‐assembled into the primary nucleus. Once the nucleates are formed, proteins can attach to existing nucleates and grow in length. The oligomers and short fibrillar seeds fragmented from elongated fibrils act as templates for the addition of monomers and other oligomers to form new fibrils. At the same time, the surface of large fibrillar seeds also promotes the branching of existing fibrils, known as secondary nucleation.^[^ ^19^ ^]^ Both elongation and secondary nucleation contribute to the steep growth phase. At last, when the system reaches a steady state, mature amyloid fibrils are formed. This structure is highly thermally stable.^[^ ^18^ ^]^ There is a significant increase in molecular interaction from dispersed protein molecules to oligomers and the primary nucleus, resulting in the formation of mature amyloid fibrils.^[^ ^20^ ^]^

### 2.2 Protein Liquid–Liquid Phase Separation

Besides the amyloid fibrils, which are stabilized by strong intermolecular interactions, proteins are also found to form liquid droplets when they are stabilized by weak molecular interactions. This process, referred to as LLPS, occurs when a homogeneous solution demixes into two distinct phases. The Flory–Huggins theory provides a preliminary explanation for LLPS by describing the Helmholtz free energy change upon homogeneous polymer–solvent mixing. The derivation of Helmholtz free energy gives a general phase diagram, showing the relationship between relative temperature and volume fraction of polymers. Below the critical temperature, protein solutions are separated into two phases via two distinct mechanisms, either by slow nucleation and growth in the binodal regium or by undergoing spontaneous decomposition in the spinodal regium (Figure 1c).^[^ ^21^ ^]^ This is the phase diagram that has been the fundamental basis of the current research on biomolecular condensates.

### 2.3 Protein Liquid‐to‐Solid Transition

The on‐and‐off weak molecular interactions make the condensates highly dynamic.^[^ ^22^ ^]^ Some protein condensates can form a glass‐like or gel‐like structure over time through the entanglement or crosslinking of the protein chains, diverging from a typical amyloid structure. (Figure 1d).^[^ ^23^ ^]^ This transition can suppress the amyloid fibril formation of aggregation‐prone proteins. For example, aggregation‐prone proteins, such as Aβ‐42 and α‐synuclein, can be sequestered in condensates to suppress their aggregations.^[^ ^24^, ^25^ ^]^

However, protein condensates are sometimes considered as the critical intermediate prior to amyloid fibril formation. Studies on several neurodegenerative disease proteins have indicated that the surfaces of condensates can effectively catalyze the conversion of liquid condensates to amyloid fibrils. This has been seen in homotypic condensates such as FUS,^[^ ^11^, ^26^ ^]^ hnRNPA1,^[^ ^12^ ^]^ α‐synuclein,^[^ ^13^, ^27^ ^]^ and TDP‐43,^[^ ^14^ ^]^ where spike‐like fibers emerge from the surface of the condensates (Figure 1e). During this process, the interfacial tension plays an important role as it leads to a significant accumulation of protein molecules at the interface between the condensates and the dilute phase. Concentrated protein molecules located at the interface are conformational expanded and are prone to have strong protein–protein interactions, thereby promoting aggregation.^[^ ^28^ ^]^ These nucleates can serve as amyloid templates to recruit protein molecules from the dilute phase and elongate to amyloid fibrils toward the exterior of condensates (Figure 1e).^[^ ^28^ ^]^ Simultaneously, the protein molecules inside the condensates diffuse to the shell of condensates and increase the thickness, leading to the gradual LST toward the center over time (Figure 1d).^[^ ^26^ ^]^

Protein LSTs can be a parallel, nonexclusive mechanism of amyloid fibril formation besides the classic nucleation of dispersed protein molecules.^[^ ^29^ ^]^ However, it is important to note that LLPS, followed by LSTs, is not the only pathway that can trigger amyloid fibril formation, even for proteins with disordered regions. Amyloid fibril formation is observed without macroscale LLPS.^[^ ^30^, ^31^, ^32^ ^]^ One of the possibilities is the existence of nanoscale condensates, which can be formed outside the known threshold of LLPS determined by mesoscale imaging techniques.^[^ ^33^ ^]^ Meanwhile, although the LSTs in this review focus on the transition into irreversible amyloid fibrils, it is also important to note the existence of reversible amyloid fibrils, where the interactions within the amyloid core are weaker and deviate from the canonical irreversible amyloid fibril.^[^ ^34^, ^35^, ^36^ ^]^ They have a more solvated structure and can potentially convert back to condensates^[^ ^37^ ^]^ or further transform into irreversible amyloid fibrils.^[^ ^38^ ^]^

We end this section by summarizing the protein phase transition products using a schematic energy landscape diagram (Figure 1f). Protein monomers can self‐assemble into various products, transitioning from higher to lower free energy states with increased molecular interactions. Oligomers represent the most disordered intermediate state, while amyloid fibrils are the most organized structure with the lowest free energy.^[^ ^39^ ^]^ We hypothesize that condensates have a lower free energy compared to oligomers as they involve a higher density of intramolecular interactions.^[^ ^40^ ^]^ The free energy of arrested condensates further decreases due to reduced dynamicity.^[^ ^41^ ^]^ Tactoids are another intermediate that is composed of short, semiflexible protein nanofibrils, displaying as nematic liquid crystals.^[^ ^42^, ^43^, ^44^ ^]^ Tactoids may have even lower free energy due to their well‐orientated structure compared to other intermediates, while their partial liquid property makes them more dynamic compared to amyloid fibrils.^[^ ^45^ ^]^

## 3 Protein Phase Transition Can Be Regulated by Their Intrinsic Factors

### 3.1 Protein–Protein Interactions Drive LLPS and LSTs

**Figure 2:**
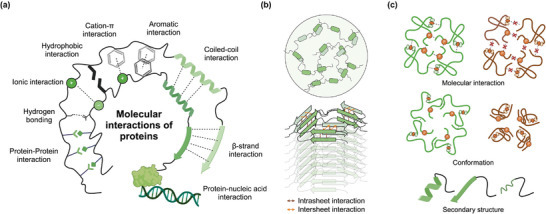
a) Common types of molecular interactions of proteins. b) Top: a sticker‐and‐spacer model of biomolecular condensates. The stickers interact and form the scaffold of condensates. The noninteracting regions or IDRs serve as spacers that are loosely packed, maintaining the dynamicity and liquid property of condensates. All kinds of molecular interactions can be found in sticker–sticker interactions. Bottom: a model of amyloid fibrils. The backbone of the fibril core is highly oriented and stabilized by both intrasheet and intersheet interactions. The intrasheet interaction is stabilized by hydrogen bonding. For irreversible amyloid fibrils, the intersheet interaction is usually stabilized by hydrophobic or aromatic interactions. c) PTMs and mutations can shift the homotypic protein LLPS and LSTs via altering protein interactions, conformations, and secondary structure.

Protein–protein interactions (**Figure** **2**a) are the key driving forces for both condensates and amyloid fibrils formations. The interactions between the functional groups of amino acids can be categorized into (Figure 2a): the ionic pairing of positive and negative charged amino acids (e.g., E–K), hydrophobic interaction (e.g., Q–N), cation‐π (e.g., Y–R, Y–K, F–R, F–K) and aromatic stacking (e.g., Y–Y, F–F), and hydrogen bonding.^[^ ^46^, ^47^, ^48^ ^]^ The condensates are liquid‐like with concentrated protein molecules undergoing dynamic interactions inside (Figure 2b, top).^[^ ^49^ ^]^ Meanwhile, amyloid fibrils are solid fibers with templated β‐sheet layers tightly packed along the fiber axis (Figure 2b, bottom).^[^ ^50^ ^]^ All the molecular interactions can be involved in forming biomolecular condensates (Figure 2b, top).^[^ ^36^ ^]^ These molecular interactions are strong enough to maintain stability, yet not too strong to allow for rapid formation and deformation, thereby preserving the dynamic nature.^[^ ^22^ ^]^ This also enables responsiveness to different external stimuli, making the protein condensate tunable biomaterials.^[^ ^51^ ^]^ After LSTs into irreversible amyloid fibrils, hydrogen bonding stabilizes the interaction within a single β‐sheet layer, whereas hydrophobic or aromatic interactions are commonly seen between the layers, forming a dry interface (Figure 2b, bottom).^[^ ^36^ ^]^ The neatly aligned molecular interactions across the amyloid fibrils and the hydrophobic core provide high stability and strong mechanical properties, enabling their use as structural biomaterials.^[^ ^52^ ^]^

As individual amino acids are combined, the conformation of the backbone shapes them into distinct secondary structures, including coiled coils and β‐strands. Notably, the coiled‐coil domains of centrosomal proteins such as centrosomin, pericentrin, and spd‐5 can effectively trigger their LLPS.^[^ ^53^, ^54^, ^55^, ^56^ ^]^ Although coiled‐coil interactions are not the key force to stabilizing amyloid structures compared to β‐sheet interactions, studies on prion domains of MED15^[^ ^57^, ^58^ ^]^ and a de novo‐designed peptide HERD‐2.2^[^ ^59^ ^]^ show that coiled coils can convert into β‐sheet structures and initiate amyloid fibril formation, suggesting that the conversion of secondary structure may be a critical mechanism for LSTs. Some condensates are also stabilized by multivalent interactions between intact protein molecules.^[^ ^60^, ^61^ ^]^ Meanwhile, multivalent protein–nucleic acid interactions are equally important to drive heterotypic phase transitions.^[^ ^62^, ^63^ ^]^

### 3.2 Intrinsically Disordered Region

Protein condensates can readily transform into solid aggregates if the molecular interactions are not restrained. The intrinsically disordered regions (IDRs) of protein molecules contribute to the flexibility of the protein backbone and the protein–solvent interactions.^[^ ^47^ ^]^ IDRs play an important role in modulating the phase behavior of biomolecular condensates. For example, the prion protein of yeast Saccharomyces *cerevisiae* Sup35 comprises an N‐terminal disordered prion domain, a central stress sensor domain, and a catalytic domain located at the C‐terminal. The N‐terminal and middle regions of the protein are more disordered compared to the C‐terminal domain. Cleavage of N‐terminal and middle domains from Sup35 leads to the formation of irreversible aggregates in vitro and gel‐like condensates in the cells, while full protein displays reversible condensation.^[^ ^64^ ^]^ This phenomenon can be rationalized using the sticker‐and‐spacer model in polymer science, where the multivalent points of the scaffold proteins and nucleic acids act as stickers to regulate the self‐assembly into biomolecular condensates, while the IDRs work as spacers to regulate the space of stickers, maintain adequate solvation volume of the condensate scaffolds and eventually keep the fluidic property of the biomolecular condensates (Figure 2b, top).^[^ ^47^, ^65^, ^66^, ^67^ ^]^

IDR domains widely exist in intracellular proteins that can undergo LLPS, such as prion‐like domains of the FUS family,^[^ ^68^ ^]^ ribonucleoprotein family,^[^ ^69^, ^70^ ^]^ RNA helicases,^[^ ^71^, ^72^ ^]^ and others. Many of these IDRs contain segments that are composed of a small subset of amino acids, not contributing to the complex structures, such as repeated sequences containing glycine (G), glutamine (Q), serine (S), and tyrosine (Y). These segments are defined as the low complexity domains (LCDs).^[^ ^73^, ^74^ ^]^

IDRs also play a crucial role in proteins that form the structural components of extracellular structures, such as spidroin (the major component of spider silk),^[^ ^75^ ^]^ mussel foot protein (the main component of mussel byssus cuticles and plaques),^[^ ^76^ ^]^ elastin (the critical protein for tissues that require stretchiness),^[^ ^77^ ^]^ and resilin (wings and jumping systems of insects).^[^ ^78^, ^79^ ^]^ These proteins are intriguing as they are composed of highly integrated repetitive motifs. For example, the mid‐block of almost all types of spidroin consists of repetitive domains enriched in glycine (G), glutamine (Q), and tyrosine (Y).^[^ ^80^, ^81^, ^82^, ^83^ ^]^ The tyrosine and glutamine residues act as stickers and form multivalent interactions, while disordered glycine repeats behave as spacers. A similar construction is also seen in the mussel byssus cuticle proteins (Mfp‐1),^[^ ^84^, ^85^ ^]^ elastin,^[^ ^86^ ^]^ and resilin.^[^ ^87^ ^]^ These highly repeated motifs display as random coils, maintaining the high solubility of native proteins in solution.^[^ ^88^, ^89^, ^90^ ^]^ They can also keep the dynamicity of protein molecules when these proteins are phase‐separated into condensates.

### 3.3 Post‐Translational Modifications

Post‐translational modifications (PTMs) have been shown to regulate the protein LLPS by altering the interaction between protein molecules and other biomolecules. So far, six main PTMs have been discovered to either accelerate or inhibit the LLPS and LSTs of proteins. These include phosphorylation, arginine methylation, citrullination, acetylation, ubiquitination, and poly(ADP‐ribosyl)ation.^[^ ^91^ ^]^ Here, we limit our discussion to protein phosphorylation, one of the most common and important PTMs.^[^ ^92^ ^]^ Phosphorylation usually occurs on serine, threonine, and tyrosine, introducing two negative charges in a phosphate group, which modifies the charge and steric properties of protein molecules.^[^ ^93^ ^]^ This change can either induce or inhibit the protein LLPS and subsequent LSTs. For example, multisite phosphorylation of FMRP LCD promotes LLPS by increasing negative charge densities and enhancing multivalent electrostatic interactions.^[^ ^94^ ^]^ Similarly, phosphorylation drives the LLPS of tau protein and α‐synuclein and their subsequent LSTs into amyloid fibrils. In contrast, non‐phosphorylated tau is incapable of LLPS, and wild‐type α‐synuclein condensates show high resistance in LSTs.^[^ ^27^, ^95^ ^]^ Interestingly, the LLPS phosphorylated C terminal of tau protein is insensitive to salt concentration, while it can be disrupted by hydrophobic interaction inhibitors like 1,6‐hexanediol. This indicates that the C terminal phosphorylation also enhances hydrophobic interactions, possibly due to conformational expansion.^[^ ^95^ ^]^ Further study showed that phosphorylation on N‐terminal sites further exposes the aggregation‐prone region of tau protein, leading to even faster LLPS and LSTs.^[^ ^96^ ^]^ By contrast, phosphorylation of the multivalent LCD of FUS protein by DNA‐dependent protein kinase reduces the LLPS and prevents LSTs.^[^ ^34^, ^97^ ^]^ Negatively charged residues prevent intramolecular collapse and intermolecular interactions via electrostatic repulsion.^[^ ^34^ ^]^ Phosphomimic mutation on the N‐terminal domain of TDP‐43 disrupts the head‐to‐tail homotypic interactions and its LLPS and LSTs.^[^ ^98^ ^]^ The impact of phosphorylation on LLPS and LSTs can be influenced by the location of the modified residue.^[^ ^99^ ^]^ Phosphorylation at sites characterized by low hydrophobicity and charge tends to unfold the locally packed structure to expanded coils, increasing disorder and thus inhibiting the LLPS and LSTs.^[^ ^100^, ^101^ ^]^ Conversely, phosphorylation near the regions with positively charged side chains will increase the valency of protein molecules due to the formation of polyampholytes, which can potentially facilitate LLPS and LSTs.^[^ ^102^, ^103^ ^]^

Phosphorylation is also the major PTM observed for extracellular proteins.^[^ ^104^ ^]^ Phosphorylation is found on serine and tyrosine residues within the repetitive domains of major ampullate silk proteins 1 and 2 (MaSp1, MaSp2). Phosphorylation is identified in proteins from natural adhesive secreta as well, such as velvet worms,^[^ ^105^ ^]^ caddisfly larvae silk glue,^[^ ^106^ ^]^ mussel byssus,^[^ ^107^ ^]^ sandcastle worm cement,^[^ ^108^ ^]^ and snail slime.^[^ ^109^ ^]^ Structural models show that phosphorylated residues help the formation of coils and α‐helixes.^[^ ^105^ ^]^ This potentially helps to solvate these natural protein molecules and prevents rapid solidification during storage. Phosphorylation can increase the electrostatic interactions with positively charged amino acid residues or divalent cations (particularly Ca^2+^ and Mg^2+^), thereby promoting the formation of complex condensates.^[^ ^110^ ^]^

### 3.4 Mutations

Mutations in the protein's primary structure will affect the molecular interactions between scaffold proteins/nucleic acids and the conformational dynamicity of the protein backbone. Both are essential to the LLPS and LSTs of proteins. For example, the mutation of FUS protein at position 156 from glycine (G) to glutamic acid (E) (G156E) was observed in patients with amyotrophic lateral sclerosis (ALS).^[^ ^111^ ^]^ From in vitro experiments, G156E FUS was found to undergo LST much faster than the wild‐type FUS protein. The mutation of glutamic acid reduces the conformational flexibility of the peptide bond and hence decreases the dynamicity of the IDR region.^[^ ^11^ ^]^ Similarly, pathological mutations of α‐synuclein at A53T and E46K trigger the LLPS and accelerate the subsequent LSTs. The native α‐synuclein potentially stays in inert conformational states. Mutations alter the structure and bring protein molecules much closer via enhanced molecular interactions.^[^ ^27^ ^]^ This is also seen in the mutated tau protein at P301L.^[^ ^95^ ^]^ On the other hand, mutation can also inhibit LLPS and LSTs. For instance, the LLPS of Q331K and M337V mutants of TDP‐43 is effectively impaired due to disruption of helix–helix interaction.^[^ ^112^ ^]^ The inhibition of LSTs can potentially lead to the change of mechanical properties of protein self‐assemblies. The mutation of human tropoelastin on R515A reduces its condensation propensity and the elasticity of fibers developed from condensates. Specifically, the mutation hinders the formation of salt bridges and the exposure of hydrophobic core, eventually leading to structural defects in self‐assembled fibers.^[^ ^113^ ^]^

In a nutshell, PTMs and mutations shift the homotypic protein LLPS and LSTs by one or a combination of the following pathways: i) changing the molecular interactions by enhancing/reducing the interaction force or the multivalency, ii) expanding or contracting protein structural conformation, and iii) altering the secondary structure of the protein to expose or hinder the aggregation‐prone region (Figure 2d). The case for a multicomponent biomolecular condensate can be much more complicated, as the varying contributions of different biomolecules to the phase behavior of entire condensates need to be investigated.^[^ ^10^ ^]^

## 4 Protein Phase Transitions Are Affected by External Stimuli

### 4.1 Temperature, Osmotic Shock, and Pressure

**Figure 3:**
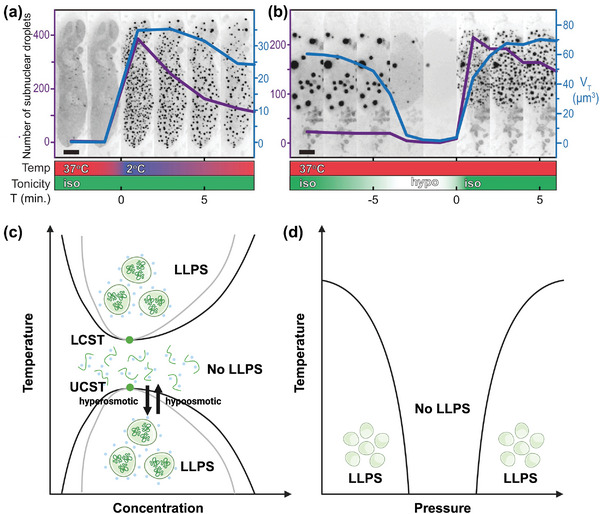
Thermoresponsiveness of biomolecular condensates. a) Ddx4 proteins expressed in HeLa cells undergo LLPS with cold shock. The purple line represents the number of condensates observed, and the blue line represents the total volume of condensates. b) Ddx4 is effectively dissolved when hypoosmotic shock is applied. When the osmotic shock toward Ddx4 is relieved, native condensates are restored. (a,b) Reproduced under the terms of the CC‐BY 4.0 license.^[^ ^114^ ^]^ Copyright 2015, Elsevier. c) The effect of temperature and osmotic shock on LLPS. d) The effect of pressure on protein LLPS. (d) Adapted with permission.^[^ ^125^ ^]^ Copyright 2019, Wiley.

Proteins can phase transit with temperature rapidly. Specifically, they can either undergo LLPS above the lower critical solution temperature (LCST) or below the upper critical solution temperature (UCST). This is an important mechanism for cells to buffer the acute temperature change. Ddx4 organelles in HeLa cells undergo rapid LLPS when the temperature shifts from 37 to 2 °C, and these condensates gradually dissolve as the temperature recovers (**Figure** **3**a). Meanwhile, if a hypoosmotic shock is applied to HeLa cells, the condensates rapidly dissolve and gradually regenerate when the system recovers to isosmotic conditions (Figure 3b).^[^ ^114^ ^]^ A recent work explores such protein condensation behavior from the perspective of water potential (the thermodynamic potential energy of water). For an ideal solution with only small solutes (such as KCl or glucose), the water potential displays a linear correlation with concentration. Based on Van't Hoff's law, the impact of temperature shock is minimized. For a solution enriched with macromolecules with high polarity or charge, such as BSA, tRNA, and polyethylene glycol (PEG), the correlation between water potential and solute concentration deviates from linearity. Thus, the water potential will be significantly affected by temperature change. This work further explores the dual effect of thermal and osmotic shocks on protein condensates using both in vitro and cell models. Specifically, acute hypoosmotic or hyperthermal conditions can effectively drive the dissolution of IDR‐containing protein condensates. Conversely, LLPS will be triggered when the temperature suddenly decreases, or an acute hyperosmotic shock is applied (Figure 3c).^[^ ^115^ ^]^ This dual thermal and osmotic effect on phase transition is potentially adaptable across many proteomes and phosphoproteomes, showing a broader physiological relevance in cellular responses to environmental stressors.^[^ ^115^ ^]^

However, not all proteins undergo LLPS at low temperatures. Several proteins, such as polyA‐binding protein (Pab1)^[^ ^116^ ^]^ and repetitive blocks from elastin, resilin, and collagen, demonstrate LCST behavior.^[^ ^117^ ^]^ The IDRs of resilin are particularly intriguing as they display both UCST and LCST.^[^ ^118^, ^119^, ^120^ ^]^ Combining sequences from elastin and resilin at different ratios results in different LCST and UCST. This suggests the influence of primary structure on proteins’ thermal responsiveness.^[^ ^121^ ^]^ The existence of LCST may be attributed to the enrichment of proline and glycine residues, which increase the overall hydrophobicity. By contrast, the enrichment of arginine‐based charged or zwitterionic motifs effectively drives UCST behavior.^[^ ^122^ ^]^

Pressure also can mediate the phase separation of proteins (Figure 3d). It has been discovered that FUS undergoes LLPS under both low (≤1 bar) and high pressure (≥2.0 bar).^[^ ^123^ ^]^ An earlier study reported that lysozyme has a similar reentrant phase separation under low and high pressure with surprisingly high thresholds.^[^ ^124^ ^]^ It is suggested that the configuration of the protein chain is unlocked when the pressure is applied, increasing its exposure to the solvent molecules. When the pressure further increases, protein molecules will pack back to achieve a smaller partial molar volume and trigger the reentrant phase separation.^[^ ^124^, ^125^ ^]^

Although there has been increasing interest in temperature/pressure‐mediated LLPS, their effect on LSTs has not been extensively studied. Previous work shows that increasing temperature from 18 to 25 °C slows down the aging of FUS condensates.^[^ ^23^ ^]^ Given that FUS can be dissolved above its UCST, we hypothesize that increasing temperature decreases the concentration gradient between the dense phase and the diluted phase. This potentially limits the diffusion of protein molecules toward the interface and inhibits the interfacial‐mediated LSTs.

### 4.2 Salt and pH

Both salt concentration and the type of salt can impact the phase behavior of protein molecules.

It has been shown that FUS and its G156E mutant, TDP‐43. Brd4, Sox2, and A11 undergo LLPS at both low and high salt concentrations.^[^ ^126^ ^]^ At high salt conditions, the hydrophobic interaction is dominant compared to the ionic interactions, promoting the LSTs. This is evident from the observation that FUS and PR_25_ condensates formed at high salt conditions are resistant to dissolution by electrostatic or polar disruptors such as polyuridine RNA and ATP.^[^ ^126^ ^]^ Similarly, tau protein undergoes reversible LLPS at both low and high salt concentrations, while at high salt concentrations, it undergoes subsequent irreversible LSTs.^[^ ^127^ ^]^

The Hofmeister series is a classification of ions based on their ability to affect the stability, solubility, and structure of proteins and other biological macromolecules,^[^ ^128^ ^]^ where kosmotropic ions interact strongly with water, stabilizing protein molecules, while chaotropic ions have the opposite effect.^[^ ^129^ ^]^ Higher concentrations of FUS and PR_25_ are required to trigger LLPS when mixed with Ca^2+^, compared to cations with less kosmotropic strength, like Cs^+^ and Na^+^. Aging of FUS and PR_25_ is also enhanced when Na^+^ is used, compared to more kosmotropic ions like Rb^+^ and Cs^+^.^[^ ^126^ ^]^ In the case of silk‐like protein, more negatively charged PO4^3−^ salts have a higher impact on LLPS compared to less charged SO4^2−^ and Ac^−^ salts.^[^ ^130^ ^]^ A more recent paper highlighted the effect of ion size on protein phase transitions, where proteins form condensates when small ions are applied.^[^ ^131^ ^]^

pH is also critical to protein phase transitions. Many proteins can undergo phase transitions around the isoelectric point due to the decrease in solubility and enhanced electrostatic interactions.^[^ ^132^ ^]^ For example, the repeat domains of tau protein K18 (pI = 9.8) are fully dissolved at pH 4.8 and can undergo LLPS when the pH value is increased to 8.8. At basic pH values, the LSTs of K18 proteins can be induced by polyanions, such as heparin, as a result of the increase of positive charge density.^[^ ^133^ ^]^ Similarly, yeast prion protein Sup35 undergoes LLPS and subsequent LSTs when pH is dropped from 7.5 to 6.0.^[^ ^64^ ^]^

Besides the effect of the charge on protein molecules, the pH value also changes their conformation, thereby affecting the phase transitions. In Sections 3.1 and 3.2, we have introduced the mid‐block repetitive domains of the spidroin as the scaffold of spider silk protein condensates and fibers. Unlike the mid‐blocks, the terminal domains of spidroin are enriched with pH‐sensitive residues.^[^ ^134^ ^]^ The N‐terminal domains undergo conformational change and can form homodimers when the pH is decreased.^[^ ^135^ ^]^ This can help the protein molecules to interconnect and lock the mid‐block scaffolds, forming a larger network.^[^ ^136^ ^]^ At the same time, the C‐terminal domain can unfold and adopt the conformation to the cross‐β sheet structure.^[^ ^129^ ^]^ These can drive the conversion of dispersed spidroin to liquid condensates and further solid fibers formation.

### 4.3 Molecular Crowding

The influence of molecular crowders on protein phase transitions is multifaceted, involving both thermodynamic and kinetic aspects. Initial studies found that the presence of molecular crowders shifts the equilibrium from unfolded to folded states, mainly due to entropic reduction caused by the excluded volume.^[^ ^137^ ^]^ Subsequent studies supplemented the contribution of enthalpic effects from protein–crowder interactions, that mainly stabilize the folded states of the protein.^[^ ^138^ ^]^ Molecular crowders also affect the degree of protein hydration. PEG as a common crowder, can promote protein–protein interaction by arresting the water molecules.^[^ ^139^ ^]^ As discussed previously, protein dehydration leads to enhanced homotypic interactions, followed by condensation and aggregation. The commonly used molecular crowders in studying protein LLPS inside cells include PEG, dextran, Ficoll, and BSA.^[^ ^140^ ^]^ They can mimic the cellular environment and promote the LLPS by decreasing the threshold concentration. For example, in vitro condensate formation of G3BP1 and wild‐type human tau protein relies on the Ficoll and PEG, respectively.^[^ ^96^, ^141^ ^]^ However, excessive molecular crowders increase the viscosity of protein condensates, which can lead to solidification. For example, high PEG concentration can result in significant solidification of FUS and α‐synuclein protein condensates.^[^ ^27^, ^142^ ^]^ This is potentially due to the further enhancement of the excluded volume effect, protein–crowder interactions, and hydrations.^[^ ^29^ ^]^ Although some research has reported that molecular crowders can suppress protein aggregation,^[^ ^143^, ^144^ ^]^ the reduction in LSTs is rarely reported for in vitro experiments. This indicates that a crowded environment can be important in recruiting biomolecules into condensates, while it may not be the key reason for suppressing the solidification of biomolecular condensates in cells.

### 4.4 Mechanical Stress

The cytoskeleton in cells forms an elastic network that provides an elastic constraint to the membrane‐less organelles, potentially affecting their number, size distribution, and density.^[^ ^145^ ^]^ A previous study has attempted to simulate this phenomenon by using oil droplets in silicone gels. It is shown that the internal pressure applied to the droplets, represented by the gel stiffness, is negatively correlated to the droplet radius. Meanwhile, the number of droplets increases with the elastic constraint and remains independent of the degree of supersaturation.^[^ ^145^, ^146^ ^]^ A more recent study further pointed out that the elasticity from the network efficiently drives and accelerates the LLPS. This can be much faster than the conventional Ostwald ripening driven by surface tension.^[^ ^147^ ^]^ In cells, the interaction between condensate and cytoskeletal proteins is much more complicated. Condensates can control the kinetics of the polymerization and build‐up of cytoskeleton filaments, and the maturation of cytoskeletal networks can also influence the dynamics of the condensates and alter their mechanical properties. This dual regulatory process can either suppress or enhance protein LLPS.^[^ ^148^ ^]^ It is suggested that birds utilize such elastic stress‐mediated protein phase transitions to generate different structural colours on their feathers.^[^ ^149^, ^150^ ^]^

Mechanical shear can impact the LSTs of biomolecular condensates. A simple example of shear generation is pipetting during the in vitro experiments. It is shown that harsh pipetting can accelerate the LSTs of FUS condensates.^[^ ^151^ ^]^ Further in vitro study explores the genericity of shear‐responsive LSTs of protein condensates into β‐sheet rich solid fibers, including FUS, Ded1, Annexin A11, z‐FF, and silk protein. Upon quantifying the shear forces required for triggering LSTs in microfluidics, this work suggests that the shear force created by axonal transport and cytoplasmic streaming may contribute to LSTs of intracellular condensates.^[^ ^52^ ^]^ Additionally, shear‐induced LSTs can be important in the fabrication of biological materials such as bioadhesives and fibers, which is demonstrated in the coming sections.

## 5 Functional Materials Derived from Protein Phase Transition Products

### 5.1 Novel Drug Discovery Strategies Targeting Protein Phase Behavior

Manipulating the behavior of biomolecular condensates has become a novel drug discovery strategy, given their close association with intercellular functions.^[^ ^152^, ^153^ ^]^ Several approaches have been proposed to develop drugs or therapeutics where certain biomolecular condensates are identified as contributing to diseases.^[^ ^153^ ^]^ For example, short‐bait RNAs^[^ ^154^ ^]^ and the small‐molecule drug mitoxantrone^[^ ^155^ ^]^ have been shown to prevent aberrant TDP‐43 condensate formation by blocking the RNA binding domains, thereby potentially blocking the pathological aggregation associated with ALS and FTD (frontotemporal dementia). Additionally, small therapeutic molecules can bind to IDRs of proteins, altering their conformational flexibility and thus affecting the dynamicity of protein molecules. Another example studies the oncoprotein composed of phase‐separation protein domain and DNA‐binding domain (PS‐DBD), which plays a role in chromosomal rearrangement in cancer cells. PS‐DBD phase separates on chromatin, followed by disrupting normal gene regulation by commandeering components from the transcription factories.^[^ ^156^, ^157^ ^]^ The DNA‐bonded PS‐DBD condensate displays a solid‐like behavior that cannot be dissolved in cells. A high‐throughput drug screening identified a small molecule drug LY2835219, which can effectively dissolve the aberrant PS‐DBD condensate.^[^ ^158^ ^]^

The process for drug discovery targeting condensates can be divided into four steps (**Table** **1**).^[^ ^153^ ^]^ The initial stage is to identify a target protein condensate with pathological implications. Bioinformatics, machine learning, and LLPS prediction tools can be used to pinpoint the potential protein sequence likely to undergo disease‐related phase transitions.^[^ ^152^ ^]^ Once the target protein is discovered, a detailed characterization of protein phase behavior can be conducted. This characterization includes studying the material properties of condensates and constructing phase diagrams considering several intrinsic and external factors mentioned in previous parts. This helps in understanding the conditions under which proteins undergo phase transitions and their stability in different environments. Following that, a two‐phase drug screening is conducted. The primary screening aims to find out the molecules that can effectively alter the phase transitions, while the secondary screening focuses on minimizing the side effects of the drugs.^[^ ^153^ ^]^ Several innovative tools have been developed to optimize the screening process. Optogenetic methods enable the light‐activatable regulation of protein phase transitions in cells by fusing the protein of interest with photo‐oligomerizable seeds.^[^ ^159^, ^160^ ^]^ This enhances the localization of intercellular protein molecules and increases the size of the condensates that sometimes are too small to observe. High‐throughput analytical methods allow the rapid analysis of a large number of samples to study the protein phase behavior in response to various drugs comprehensively.^[^ ^161^ ^]^ Microfluidic technology enables the precise control of fluids in small volumes, providing a great platform to monitor protein phase transitions and detect the partition of small molecules in condensates with high reproducibility and low material consumption.^[^ ^162^ ^]^

**Table 1:** A general flowchart for drug discovery targeting protein phase transitions.^[^ ^153^ ^]^

Step	Objective	Potential techniques
Hypothesis	Target aberrant condensation linked to disease Target the formation of biomolecular condensates in an identified disease mechanism	Bioinformatics and machine learning^[^ ^163^, ^164^ ^]^ LLPS prediction database^[^ ^165^ ^]^ Molecular dynamics simulation^[^ ^166^ ^]^
Characterization	Material properties of condensates A phase diagram showing the shifting phase behavior based on intrinsic factors, external stimuli, and interaction with other proteins/nucleic acids.	Confocal microscopy and other advanced microscopy techniques^[^ ^156^ ^]^ High‐throughput systems for imaging and analytical assays^[^ ^161^, ^167^ ^]^ Microfluidics^[^ ^162^ ^]^ Optogenetics^[^ ^159^, ^160^ ^]^
Primary screening	Screening of drug that fits the target (e.g., dissolution/formation of a condensate, change the material property)	
Secondary screening	Minimize cytotoxicity and off‐target effects Optimize the drug partitioning inside the target condensate	

### 5.2 Protein Condensates as a Carrier in the Drug Delivery System

**Figure 4:**
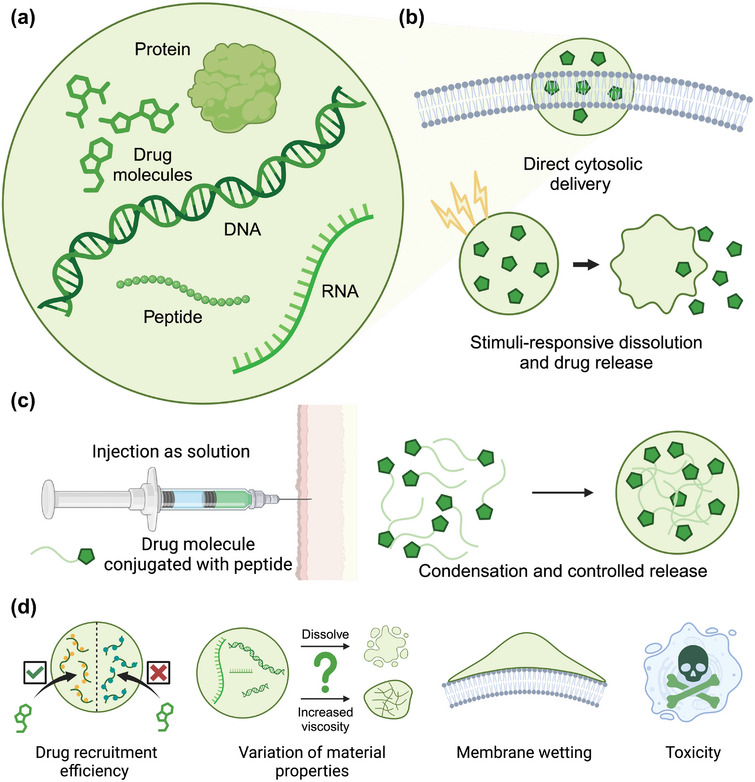
a) Protein condensates can serve as carriers for drug molecules, proteins, peptides, and nucleic acids. b) Intracellular drug delivery pathway. Protein condensates carrying therapeutics can potentially transport through the membrane directly without the endocytosis, followed by stimuli‐responsive dissolution of cargoes and release of drugs. c) Protein drugs can also be injected as a liquid, followed by temperature‐mediated LLPS inside the human body. The condensates formed can control the release kinetics and protect the drugs from degradation. d) Design considerations of protein condensate as drug delivery vesicles.

Protein/peptide condensates can serve as carriers for delivering drug molecules and nucleic acids into the human body, leveraging their liquid‐like properties to recruit and preserve the bioactivity of cargoes under aqueous conditions (**Figure** **4**a).^[^ ^168^ ^]^ In intracellular drug delivery, protein condensates can bypass the classical endocytosis and transport the cell membrane via direct cytosolic delivery, which is a more efficient and energy‐independent process.^[^ ^15^ ^]^ Meanwhile, the stimuli‐responsiveness nature of protein allows for the controlled release of cargoes.^[^ ^169^ ^]^

Drug‐containing condensates can be directly delivered into the body, which then releases the drug cargoes upon dissolution (Figure 4b). One notable example is HBpep, a bioinspired short peptide based on histidine‐rich beak proteins. HBpep peptide has five repeats of sequence GHGXY but with a lysine residue inserted at position 16. The amine group of lysine residue is conjugated with disulfide‐containing moiety, connected with an extra acetyl or phenyl group, to regulate the dissolution rates. The GHGXY repeats display a crafted sticker‐and‐spacer design, where glycine provides the flexibility of the backbone region, and tyrosine behaves as stickers via π‐stacking. The histidine and lysine residues make this peptide‐based condensates pH‐responsive. The conjugation of extra moiety shifted the critical LLPS pH to 6.5, while cleavage of this motif results in a higher LLPS threshold of pH 9.0. This composition allows the controlled LLPS of HBpep. Under physiological pH conditions, this peptide is capable of forming condensates for delivering mRNA and various proteins or peptides into HepG2 cells. Intercellular reducing agents can cleave the desulphated moiety, dissolve peptide‐based condensates via shifting the LLPS threshold, and release the contained drugs. Such a delivery approach can skip the classical endocytosis and directly penetrate the cell membrane, which is energy‐independent and has fast uptake kinetics.^[^ ^15^ ^]^ However, the mechanisms underlying direct cytosolic transport remain complex and not fully understood.

Another delivery approach is to inject a solution of proteins/peptides with therapeutic molecules and trigger condensation inside the human body (Figure 4c). This approach utilizes the formation of condensates to control the release of the therapeutics and protect the drug molecules from degradation. Elastin‐like peptide‐based condensates, known for their LCST behavior, are particularly well suited for this application. These peptides remain injectable solutions under low temperatures and transit into condensates when body temperature is reached. For instance, elastin‐like peptide conjugated with glucagon‐like peptide 1 (GLP1) can undergo temperature‐mediated LLPS inside the body. GLP1 is a receptor for treating type 2 diabetes but is highly unstable. Encapsulating GLP1 within elastin‐like condensates extends its lifetime and circulation duration, verified in both mouse and monkey models.^[^ ^170^ ^]^

To further refine the drug delivery strategy, we propose several considerations in selecting suitable protein condensates as carriers that are crucial in developing efficient and safe drug delivery systems (Figure 4d).

**Recruitment efficiency of drug molecules**. The recruitment efficiency of drug molecules varies depending on the intrinsic scaffolds to form condensates. For example, MED1 is a multifunctional protein that could form condensates under physiological conditions, exhibiting high partitioning for drugs like cisplatin and mitoxantrone.^[^ ^156^, ^171^ ^]^ However, when all aromatic amino acids are removed by mutation to alanine, the droplets lose their ability to effectively recruit the small molecules.^[^ ^171^ ^]^ This suggests that electrostatic interactions through aromatic ring interactions could be the main mechanism of drug molecule partitioning.

**Variation of material properties**. The material properties of protein condensates can change significantly after the recruitment of therapeutics, especially for nucleic acids. The viscoelasticity and stability of the condensates can be highly influenced by the concentration and length of nucleic acids. High RNA concentrations can dissolve protein condensates due to electrostatic charge screening.^[^ ^172^, ^173^ ^]^ A simulation study suggests that short RNAs are more potent in driving protein phase separation, while longer RNAs increase the viscosity of the condensate.^[^ ^174^ ^]^ Therefore, the selection of nucleic acids as cargoes requires careful consideration beyond mere partitioning to include the viscosity and fluidity of the final condensates, which are crucial for targeted drug delivery.^[^ ^175^ ^]^

**Condensate–membrane interaction**. Due to the liquid‐like property of biomolecular condensates, their interaction with cell membranes behaves differently based on the salinity of the environment, membrane surface charge, and many other factors. For example, altering the surface charge of giant unilamellar vesicles significantly affects the wetting property of glycinin condensates on them.^[^ ^176^ ^]^ Further work is required to investigate the impact of membrane wetting on drug uptake kinetics and efficiency.^[^ ^177^, ^178^ ^]^

**Toxicity**. The toxicity of drug carriers could arise from several factors, such as off‐targeting effects and low biodegradability. Proteins and peptides are inherently degradable by cellular mechanisms, converting them into amino acids by proteasomal activities^[^ ^179^ ^]^ ranging from minutes to 48 h in vivo.^[^ ^180^, ^181^ ^]^ Peptide‐based carriers have been reported to have high biodegradability and prolonged life span after intracellular delivery.^[^ ^182^ ^]^ However, the cytotoxicity of the protein/peptide‐based condensates highly depends on their reactivity and solubility in cells and requires further investigation.

### 5.3 Controlled Biomineralization by Protein Condensates

Protein molecules can alter the morphology and properties of inorganic materials by forming heterogenic condensates. This process can control the kinetics of mineral growth, leading to the formation of ordered hierarchical structures. This organic–inorganic interplay is fundamental to biomineralization, which is essential to various bone and shell structure generation. Although the major components of biominerals are inorganic materials,^[^ ^183^ ^]^ the organic parts significantly improve the mechanical properties of the composites up to two orders of magnitude.^[^ ^184^ ^]^ Here, we discuss how protein condensates, as the organic subparts, can affect the growth of biominerals.

**Figure 5:**
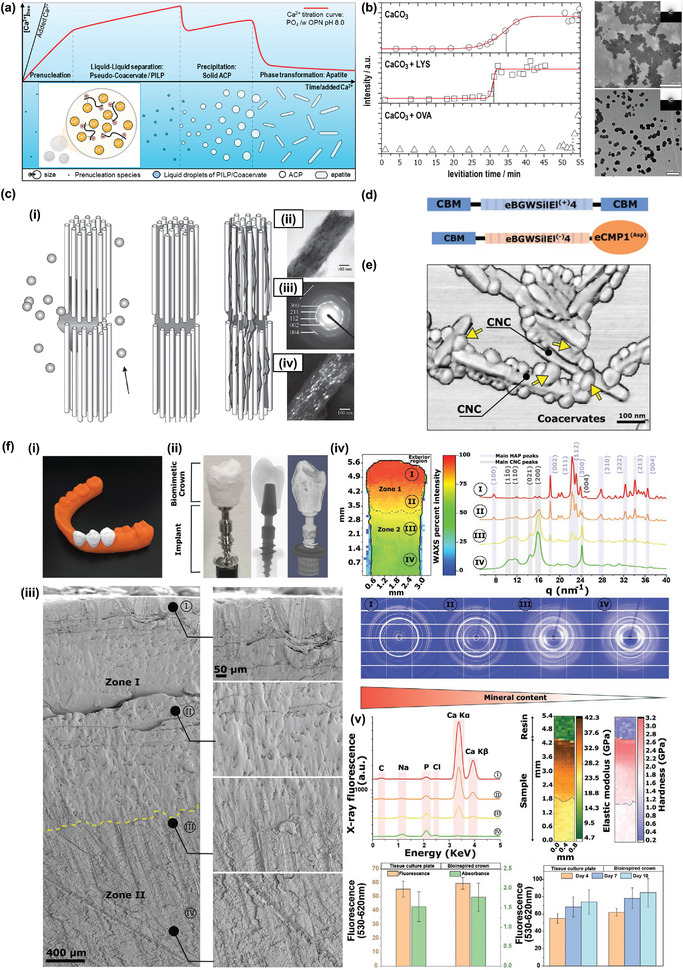
Biomineral formation is regulated by protein condensates. a) Calcium concentration trends in the presence of the PILP process. The precrystallization intermediates are stabilized by protein via multivalent interactions, delaying the precipitation and biomineralization. Reproduced with permission.^[^ ^194^ ^]^ Copyright 2016, American Chemical Society. b) The effect of lysosome and ovalbumin on the kinetics of calcium carbonate mineralization and final morphology (upper: lysozyme, bottom: ovalbumin). Reproduced with permission.^[^ ^191^ ^]^ Copyright 2011, American Chemical Society. c) (i) A schematic illustration of the PILP process collaborating with collagen fibril. Amorphous calcium phosphate stabilized by negatively charged protein/polymer adheres to the positive‐charged collagen fibril and infiltrates into the gaps of the fibril. The crystallization of calcium phosphate forms lamellar crystals following the direction of collagen fibril. (ii) Bright field‐TEM image of a single mineralized collagen fibril. The selected area diffraction pattern (iii) and reflection in dark‐field TEM (iv) reveal the orientation of minerals inside the collagen fibril. Reproduced with permission.^[^ ^196^ ^]^ Copyright 2016, Wiley. d) The silk‐inspired protein construct used in the case study. The upper one is used to reinforce CNC scaffolds. The downer one is used for calcium biomineralization. e) The 3D‐rendered topographic AFM image of protein–CNC composites. f) (i) Bioinspired premolar implants settling in the 3D‐printed human low jaw. (ii) Premolar implant composed of a titanium screw and biocomposite crown. (iii) SEM images from the exterior to the crown's interior. The distinct morphology of biominerals and protein–CNC scaffolds can be seen. (iv) Synchrotron 2D WAXS mapping of the regions in (iii), the main peaks of CNC appear at the bottom layers of the crown, and the peaks of apatite crystals display at the top layers of the crown. This is further supported by X‐ray fluorescence spectra in (v). The AB and LDH assay of adult human dermal fibroblast confirmed the cell viability of the bioinspired crown. (d–f) Reproduced with permission.^[^ ^198^ ^]^ Copyright 2021, Wiley.

Calcium carbonate and calcium phosphates are common inorganic components of biominerals. The growth of calcium carbonates was believed to follow the classical nucleation theory.^[^ ^185^ ^]^ In recent decades, increasing evidence shows the formation of metastable, amorphous nanosized precursors via LLPS before crystallizing.^[^ ^186^ ^]^ Further research indicates that these precursors can be stabilized by charged polymers to prevent premature crystallization, which is known as a polymer‐induced liquid precursor (PILP) process (**Figure** **5**a).^[^ ^187^ ^]^ Native negatively charged proteins enriched with IDRs can manipulate crystallization kinetics via multivalent ionic interactions and forming heterotypic biomolecular condensates, while positively charged proteins have the opposite effect. Research on egg white protein has shown that negatively charged ovalbumin can lag the crystallization of calcium carbonate, while positively charged lysozyme can accelerate the mineralization (Figure 5b).^[^ ^188^, ^189^, ^190^ ^]^ During eggshell formation, ovalbumin binds to Ca^2+^ and forms condensates, which can stabilize the amorphous state and slow down the crystallization, leading to an ordered hierarchical assembly of eggshells with good mechanical properties.^[^ ^191^, ^192^ ^]^ A similar observation is also seen in a study on osteopontin (OPN), a negatively charged protein in mammalian bone and teeth. OPN displays a high calcium binding affinity, with ten binding sides per protein molecule. This is potentially contributed by multiple phosphorylated residues and acidic amino acids.^[^ ^193^ ^]^ Protein condensates can recruit calcium cations via protein–cation interaction, effectively stabilizing the amorphous state of calcium salt and delaying its crystallization into apatite.^[^ ^194^, ^195^ ^]^ Although OPN is not the dominant protein in the organic matrix of bones, it plays a critical role in directing the controlled crystallization of hydroxyapatite alongside the fibrillization of collagen via the PILP process (Figure 5c).^[^ ^196^ ^]^

The concept of controlled biomineralization via the PILP process has been applied to generate artificial teeth implants. A designed triblock protein composed of an intrinsically disordered domain from silk fibroin and elastin with cellulose‐binding terminals is used (Figure 5d). This protein can undergo LLPS to densely pack the cellulose nanocrystal (CNC) rods and increase the degree of orientation inside condensates, resulting in a nematic framework with high rigidity (Figure 5e). Remarkably, this condensate–CNC scaffold shows minor brittleness, as the protein condensates glue the CNC rods together, creating a seamless and robust structure.^[^ ^197^ ^]^ Furthermore, another protein with a mineralizing domain replacing the original cellulose binding motif is designed (Figure 5d). The condensates of this protein are loaded on the top of the developed CNC‐composite networks, allowing the accumulation of cations and apatite mineralization within the scaffold. Controlling the concentration of mineralizing motifs can regulate the degree of mineralization, resulting in a stiffness gradient from the exterior to the interior of the scaffold. This enables the development of dental implant materials that resemble the condition of human teeth (Figure 5f).^[^ ^198^ ^]^

### 5.4 Protein Condensate Microreactors for Biosynthesis

Protein condensates, such as nucleoli, Cajal bodies, and RNP bodies, can act as reaction crucibles to enhance biological reaction rates.^[^ ^9^ ^]^ They provide a platform for effectively recruiting the reactants while strongly excluding the products, reaction inhibitors, and competitors.^[^ ^199^ ^]^ Furthermore, the apolar, charge‐dense, and locally crowded milieu reduces the activation energy and/or Gibbs free energy required for the reaction.^[^ ^200^, ^201^ ^]^ It also helps maintain a stable reaction environment with low water activity and effective pH, isolated from the diluted phase.^[^ ^199^, ^202^ ^]^

**Figure 6:**
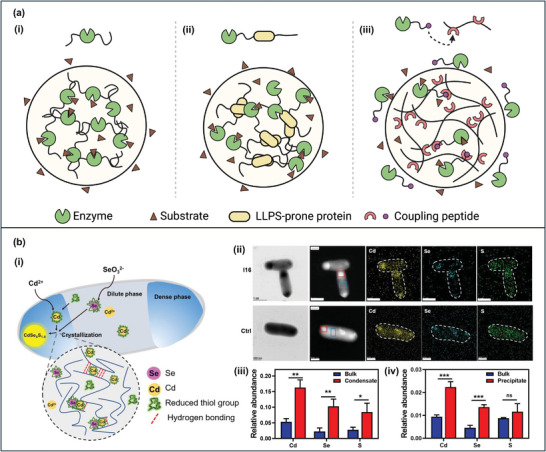
a) Design strategies of protein condensates as microreactors for enzymatic reactions. (i) Some enzymes can undergo LLPS by themselves, recruiting substrate molecules inside and catalyzing reactions. (ii) Nonphase separating enzymes can be fused to LLPS‐prone proteins to form condensates and improve catalytic efficiency. (iii) Pairs of coupling peptide motifs can be fused to enzymes and protein condensate scaffolds. This allows the effective recruitment of enzymes into engineered protein condensates. b) Protein condensate microreactor for QD synthesis. (i) A schematic diagram of CdSe*_X_* S_1−_ *_X_* QDs synthesis inside cells aided by biomolecular condensates. (ii) CdCl_2_ and Na_2_SeO_3_ are effectively recruited by silk protein condensates expressed in cells and form QDs, while only precipitates with irregular morphology are observed in control cells. (iii, iv) The abundance of Cd, Se, and S elements are significantly different within the outside of the condensates in silk protein‐expressing cells (iii), while the S element within the outside of the participation in control cells is insignificant (iv). This implies the crucial role of biomolecular condensates in the recruitment of cysteine and the synthesis of QDs. Reproduced with permission.^[^ ^207^ ^]^ Copyright 2022, Wiley.

It has been of particular interest how such properties of protein condensates can be utilized as microreactors for enzymatic reactions. Some enzymes themselves can undergo LLPS and recruit the reactants (**Figure** **6**a(i)). For example, horseradish peroxidase (HRP) and glucose oxidase (GOx) are enzymes that can oxidize both glucose and substrate, and they are frequently used together for glucose detection. Both enzymes can form condensates in a crowded environment. After LLPS, the catalytic efficiency increases by around two orders of magnitude, potentially contributed by both conformational changes of the enzymes and the high concentration of localized reactants.^[^ ^203^ ^]^ Enzymes incapable of LLPS can be fused to LLPS‐prone proteins to form enzymatic microreactors (Figure 6a(ii)). The modified enzymes with a higher propensity for LLPS are more effective at recruiting substrates, which in turn boosts catalytic efficiency.^[^ ^204^ ^]^ Enzymes can also be fused to LLPS‐prone proteins tagged with photoresponsive oligo seeds, allowing the enzymatic reaction to be regulated by light‐controllable LLPS.^[^ ^205^ ^]^ Another option is to use pairs of interacting peptide motifs to enable the recruitment of enzymes and other guest biomolecules into host protein condensates (Figure 6a(iii)). For instance, RIDD‐tagged MenF and MenH enzymes are better recruited to RIAD‐tagged Shank/Homer condensates, leading to a higher production rate than in the untagged system.^[^ ^206^ ^]^

Besides enzymatic reactions, protein condensate microreactors can also be utilized to synthesize inorganic products such as quantum dots (QDs). Spider dragline silk protein condensates located in silk protein‐expressing *Escherichia coli* cells can recruit Cd^2+^, SeO_3_^2−^, and sulfur‐containing reducing agents to synthesize CdSeS_1−_ *_x_* QDs (Figure 6b). It is proposed that such biosynthesis is possibly a mechanism of alleviating the cytotoxicity of the heavy metal ions, as the wide spatial distribution of synthesized QDs throughout the bacteria cell avoids the local accumulation and precipitation of metal ions. The protein condensates play two important roles in this biosynthesis. It acts as a reaction crucible to concentrate the heavy metal ions and sulfur‐containing reducing molecules in cells, supporting the synthesis of QDs. It also serves as an organizational hub to sequester and stabilize nascent QDs, apart from other intercellular components. This improves the fluorescence intensity and lifetime of QDs, promising their usage in subcellular imaging.^[^ ^207^ ^]^

### 5.5 Protein Condensate‐Based Bioadhesives

**Figure 7:**
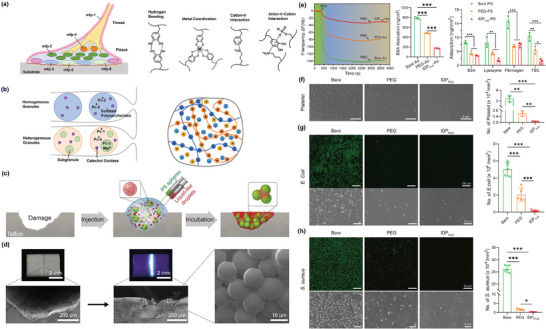
Mussel foot protein and sandcastle worm protein‐based bioadhesives. a) Left: distribution of major Mfps in the plaque of mussel byssus. Right: typical molecular interactions that stabilize Mfp condensates. b) The composition of protein in the cement of sandcastle worms, stored in with‐membrane granules. When the membrane is ruptured by mechanical shear, complex condensates are formed by the components from both granules. (a,b) Reproduced with permission.^[^ ^231^ ^]^ Copyright 2023, Elsevier. c) Schematic illustration of damage repair on a Teflon substrate. Condensates and PS spheres are injected simultaneously into the damaged site. The PS spheres are glued to the surface by aged condensates upon 12 h incubation at 4 °C. d) The photographs of the Teflon substrates before and after repair. The UV light revealed the adhesion of protein condensates. SEM images showed the PS spheres are tightly glued together in the damaged site. (c,d) Reproduced with permission.^[^ ^221^ ^]^ Copyright 2019, AAAS. e) Quartz crystal microbalance with dissipation (QCM‐D) curves showing the engineered‐FUS bioadhesive‐modified Au and PS surfaces have lower adsorption of BSA, lysozyme, fibrinogen, and FBS, compared to bare or PEG‐treated surfaces. f–h) Bioadhesive‐treated silicon surfaces have lower adsorption of platelet (f), *E. coli* (g), and *S. aureus* (h) compared to bare and PEG‐coated surfaces. (e–h) Reproduced with permission.^[^ ^222^ ^]^ Copyright 2022, American Chemical Society.

If you walk near the ocean, you may discover mussels adhering tightly to the rocks, resisting the drag forces from the sea waves. The mussel foot protein (Mfp) from mussel byssus is responsible for such strong adhesion. The mechanism inspires the design of underwater adhesive materials. The main types of Mfp (Mfp‐1 to 6) are all enriched with phenylalanine, tyrosine, and DOPA (3,4‐dihydroxyphenylalanine, post‐translated from tyrosine) residues.^[^ ^208^, ^209^ ^]^ DOPA molecules are active for various molecular interactions, including hydrogen bonding, metal coordination, hydrophobic interactions, and ionic‐π interactions, allowing Mfp to adhere to different types of surfaces (**Figure** **7**a).^[^ ^210^ ^]^ Mfps are synthesized inside the glands of mussels and then stored within plaque secretory vesicles under acidic conditions as liquid condensates. This condensate formation is likely contributed by the hydrogen bonding between DOPA (3,4‐dihydroxyphenylalanine) residues.^[^ ^211^ ^]^ As these vesicles are transported along the longitudinal ducts extended from the plaque gland, they mix with metal‐storage particles under a gradually increasing pH gradient. This environment fosters the crosslinking of Mfp with metal ions (such as Ferrum, vanadium, and aluminum) via DOPA–metal bonds. This process results in the porous mussel plaque, which is extruded with mechanical shear and extensional flow at the outlet of the ducts.^[^ ^212^, ^213^, ^214^ ^]^ The high oxygen content and positive redox potential of seawater can make DOPA prone to oxidize and reduce the adhesivity of mussel plaque. The thiol group‐rich Mfp analogs Mfp‐6 and Mfp‐16 to 19 can address this issue by reducing the oxidation.^[^ ^79^, ^215^ ^]^ In Mfp condensates, Mfp‐3 and Mfp‐5 are responsible for interfacial adhesion plaque and surface, whereas Mfp‐6 serves as chemical energy reservoirs to resist oxidation into dopaquinone and maintain the long‐term adhesion to mineral or oxide surfaces.^[^ ^216^ ^]^

Sandcastle worm is another species widely studied for developing adhesive materials (Figure 7b). The adhesive, sometimes called cement, from the sandcastle worm *Phragmatopoma californica* comprises two types of granules. One is composed of polybasic proteins, including Pc2, Pc5, and sulfated polysaccharides. The other contains polyampholytic proteins (Pc3A), polyphosphate proteins (Pc3B), polybasic proteins (Pc1, Pc4), and Mg^2+^ ions.^[^ ^217^ ^]^ Catechol oxidase is in both granules to trigger the crosslinking of DOPA side chains.^[^ ^218^ ^]^ The crosslinking occurs when the fluids from both types of granules are mixed and undergo LLPS. The catechol effectively oxidizes the DOPA into dopaquinone, which can then be crosslinked by copper ions.^[^ ^218^ ^]^ Meanwhile, the pH change from the secretory system (pH < 6) to the seawater (pH > 8), as well as the ionic replacement from Mg^2+^ to Ca^2+^ induce the LSTs of the complex condensates to allow the self‐assembly of sticky structures.^[^ ^219^ ^]^

Recombinant technology enables us to reconstruct the crafted sequence of Mfp and develop adhesive biomaterials mimicking the nature of mussels. The adhesion of engineered Mfp protein is achieved by the formation of condensates and surface wetting, followed by LSTs into a uniform coating with enhanced cohesiveness triggered by the addition of salts or altering pH.^[^ ^211^ ^]^ It is worth noting that as *E. coli* expression cannot replicate the natural PTMs of tyrosine, the recombinantly produced Mfp typically requires mushroom tyrosinase to convert tyrosine to DOPA.^[^ ^220^ ^]^ Interestingly, it is reported that recombinant Mfp constructs without PTMs into DOPA fail to undergo LLPS in vitro, which highlights the critical role of enhanced hydrogen bonding brought by PTMs.^[^ ^211^ ^]^ Mfp‐inspired protein bioadhesives can be used to repair the damaged surfaces. A synthetic Mfp5 protein fused to LCD of TDP‐43 displays a high underwater adhesivity on various surfaces, including polytetrafluoroethylene (PTFE, “Teflon”) and polyethylene terephthalate (“Mylar”) (Figure 7c,d). The fusion of TDP‐43 LCD relieved the dependence of Mfp phase transitions on pH and salt concentration, enabling adhesion under broad conditions.^[^ ^221^ ^]^ Mfp‐inspired protein bioadhesives have also been used to deal with biofouling and foreign body response, which are severe challenges in biomedical implants. The accumulation of collagen, cells, and microorganisms may lead to a strong inflammatory response and threaten the patient's life. The surface of implants can be coated with protein condensates, creating long‐lasting functional surface adhesives. Specifically, polystyrene (PS) or silicon modified with dopamine–maleimide peptides allows the attachment of engineered FUS protein condensates with additional cytidine groups at the N terminals. The composite adhesive displays outstanding behavior in mitigating biofouling and the host's foreign body response when coated onto implant surfaces (Figure 7e–h). Furthermore, the bioadhesive coating significantly reduces adhesion for platelets, *E. coli* cells, and *Staphylococcus aureus* cells in vitro and lowers the inflammatory chance in vivo.^[^ ^222^ ^]^

The idea of complex coacervation from sandcastle worms is also involved in designing protein condensate‐based bioadhesives. Hyaluronic acid (HA) is an anionic glycosaminoglycan commonly used to form complex condensates with recombinant mussel foot proteins to buffer cationic amino acids such as lysine and arginine.^[^ ^223^ ^]^ It is a natural product in the extracellular matrix, tissues, and body fluids with considerable biocompatibility and degradability.^[^ ^224^ ^]^ The high underwater adhesion of the mussel foot and sandcastle worm proteins also makes them potential biomaterials for suturing wounds exposed to bulk body fluids. Complex condensates made of recombinant mussel adhesive protein and HA display high potential in the sealant of urinary fistula. The adhesive strength of such designed biomaterial is comparable to conventional products such as cyanoacrylate derivates and fibrin glue. It displays a more prolonged adhesiveness when facing the pressure inside the bladders. Also, the protein‐based nature of the condensates significantly reduces the issue of toxic degradation products and allergic reactions.^[^ ^225^ ^]^ Another work has used a similar construct to encapsulate deproteinized bovine bone minerals (DBBMs). The DBBM–protein condensates stabilize the bone graft cluster, improve osteoconductivity, and promote bone regeneration.^[^ ^226^ ^]^ HA–protein condensates stabilized by opposite charges can be extended to even broader protein sources, including lysozyme,^[^ ^227^ ^]^ silk fibroin,^[^ ^228^ ^]^ and nonprotein sources like chitosan.^[^ ^229^ ^]^ Meanwhile, the chemistry of DOPA‐induced adhesion and complex coacervation using opposite‐charged polyelectrolytes have inspired the design of polymer‐based condensates.^[^ ^230^, ^231^ ^]^

### 5.6 Fiber Spinning Exploiting Protein Liquid‐to‐Solid Transition

Fiber spinning from spiders, silkworms, velvet worms, mussels, hagfish, and slimes are classic natural examples of fabrication via LSTs.^[^ ^16^ ^]^ Here, we limit our discussions to silk fiber production, the most intuitive and widely studied example in recent decades. We want to highlight that forming condensates and semicrystalline structures can serve as a quality control parameter, potentially critical to improving the mechanical properties of artificial silk fibers.

**Figure 8:**
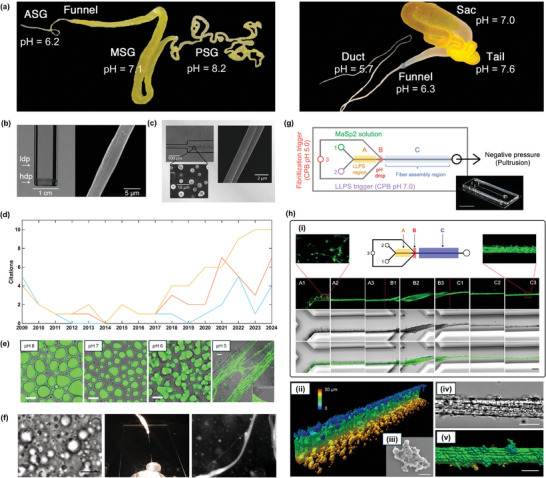
Biomimicking silk spinning. a) The configuration of silkworm and spider silk glands and the pH distribution. Reproduced under the terms of the CC‐BY 4.0 license.^[^ ^234^ ^]^ Copyright 2016, MDPI. b) Left: LLPS of eADF3 into low‐ and high‐density phase (ldp/hdp). Right: SEM image of silk fiber drawn from hdp. Reproduced with permission.^[^ ^244^ ^]^ Copyright 2007, Wiley. c) Microfluidic experiments on eADF3 revealed that elongational flow and mechanical drawing are critical for fiber formation. Otherwise, spherical aggregates are formed. Reproduced with permission.^[^ ^246^ ^]^ Copyright 2009, Wiley. d) Citations of ref. [244] (red), ref. [245] (blue), and ref. [246] mentioning the keyword “liquid–liquid phase separation” from 2009 to 2024. e) pH responsiveness of recombinant MaSp2. Reproduced with permission.^[^ ^247^ ^]^ Copyright 2020, AAAS. f) Shear‐responsiveness of regenerated silk protein with dextran. A fiber can be mechanically drawn from the condensate solution. Reproduced with permission.^[^ ^52^ ^]^ Copyright 2020, Springer Nature. g) Microfluidic design for mimicking the mechanical shear and pH gradient in silk fiber spinning. h) Morphology of MaSp2 in microfluidic channel. (i) Brightfield and fluorescent image of recombinant MaSp2 in different channel sections. (ii) 3D reconstruction of condensates along the channel. (iii) SEM image of silk protein droplets. Scale bar: 10 µm. (iv) Bright field and (v) 3D reconstruction of recombinant MaSp2 protein fiber in section C3, displaying a hierarchical structure. Scale bar: 5 µm. (g,h) Reproduced under the terms of the CC‐BY 4.0 license.^[^ ^248^ ^]^ Copyright 2024, Springer Nature.

Dragline silk from spiders and *Bombyx mori* silkworms are of the most interest due to their superior mechanical properties. Fibroins and spidroins are stored at the posterior silk glands in silkworms and major ampullate silk glands in spiders, respectively, as liquid crystals, condensates, or micelles.^[^ ^232^, ^233^ ^]^ The decreasing pH gradient, shear, and extensional flow along the gland initiate the solidification of silk protein, elongate the semicrystalline structure of silk protein, and eventually form straight fibers from the thin anterior gland or duct (**Figure** **8**a).^[^ ^234^ ^]^

Existing artificial silk fiber spinning relies on two methods for sourcing the silk protein. The first pathway is to dissolve the natural silk protein, such as *Bombyx* mori cocoon or *Nephila clavipes* spider silk fiber. The typical solutions to dissolve natural silk sources include lithium bromide (LiBr), hexafluoroisopropanol, and others.^[^ ^235^, ^236^ ^]^ Specifically, silkworm cocoon pieces are degummed by boiling to remove the sericin, as sericin will induce the aggregation of the regenerated silk. The mixture is dried and fully dissolved in 9.3 m LiBr, followed by dialysis and centrifugation, resulting in a clear aqueous silk solution with a molecular weight of around 100 kDa.^[^ ^235^ ^]^ Silk stock formed by this pathway is referred to as regenerated silk protein.

The second pathway is reproducing the silk protein via recombinant protein production technology. The critical step of the process is gene cloning of the desired spidroin, fusing the sequence to the expression vector, and transforming the plasmid into the suitable expression host. The recombinant protein produced can be harvested from the positively transformed cells, followed by purification and validation via analytical techniques. However, the repetitive sequence with high molecular weight and overall structural complexity has been a great challenge over decades. Significant efforts, such as tDNA upregulation^[^ ^237^, ^238^ ^]^ and changing the host system from *E. co*li to other species^[^ ^239^, ^240^, ^241^ ^]^ have been made to tackle the host's low tolerance to repetitive sequences, resulting in a low level of expression and stability. The current protein expression technology allows the production of MaSp protein (recombinant silk protein) up to 285–556 kDa.^[^ ^79^, ^238^ ^]^

Both regenerated and recombinant silk proteins can be directly used for fiber spinning. To date, several spinning methods have been developed, including dry‐spinning, wet‐spinning, electrospinning, direct drawing, and direct writing.^[^ ^242^ ^]^ Although these spinning methods have been trying to mimic natural silk spinning, the final mechanical properties of the fibers are still far away from the natural dragline silk.^[^ ^243^ ^]^

It has been demonstrated that condensates are a precursor for silk fiber formation using both regenerated and recombinant silk protein. An early study in 2007 developed an engineered silk protein eADF3, inspired by the *Araneus diadematus* spider silk. eADF3 can undergo LLPS forming condensates when the pH is decreased to 6 in the presence of kosmotropic salts. These condensates display an LCST at 25.4 °C, and silk thread can be drawn from the condensates (Figure 8b).^[^ ^244^ ^]^ The experiment was repeated in 2008 using the microfluidic device, which further confirmed condensates formation and the dominant effect of the shear to convert the condensates to extended fibers (Figure 8c).^[^ ^245^, ^246^ ^]^ Since 2017, due to the increasing interest in pathological LLPS and LSTs, there has been a significant growth in the attention to these results (Figure 8d). In 2020, using recombinant technology, it was discovered that MaSp can undergo LLPS when mixing with potassium phosphate under neutral pH (pH 7–8). When pH reaches ≈6, the protein condensates rapidly solidify into a gel‐like state (Figure 8e).^[^ ^247^ ^]^ Such pH sensitivity fits native regenerated spidroins from *T. clavata* spiders. This work also confirmed the critical role of repetitive central blocks together with N and C terminal domains in driving LLPS.^[^ ^247^ ^]^ Another result supplemented the effect of kosmotropic salts on the LLPS and aging of the recombinant spider silk condensate by using a modified eADF3 protein.^[^ ^130^ ^]^ Meanwhile, it was reported that regenerated silkworm protein can also undergo LLPS with dextran as the molecular crowder. Silk fiber can be generated by direct drawing from liquid condensates (Figure 8f).^[^ ^52^ ^]^ Most recent work used a microfluidic device and recombinant MaSp2 protein to mimic the fiber spinning (Figure 8g). Compared to the previous design, this work applies an extra channel to trigger the pH drop toward the condensates transported from the mixing region. The conversion of condensates to fibrils under shear can be visualized from images of channel subparts (Figure 8h).^[^ ^248^ ^]^ These findings suggest that LLPS could be a potential intermediate stage during silk fiber spinning. LLPS can be a promising quality control standard during the silk spinning process. It will help to better mimic the natural spinning process and improve the properties of silk fiber products.

### 5.7 Controlled Microtubule Fabrication

Microtubules are the major components of the eukaryotic cytoskeleton and determine the structure and shape of eukaryotic cells. They also direct the movement of motor proteins in cells, such as dynein and kinesin, to transport intracellular cargoes,^[^ ^249^ ^]^ which is an important mechanism in cell mitosis and motility.^[^ ^250^ ^]^ In recent decades, microtubules have also been used as building blocks of nanoelectronics devices,^[^ ^251^ ^]^ nanomaterials,^[^ ^252^ ^]^ and bio–micro/nanorobots.^[^ ^253^, ^254^ ^]^

**Figure 9:**
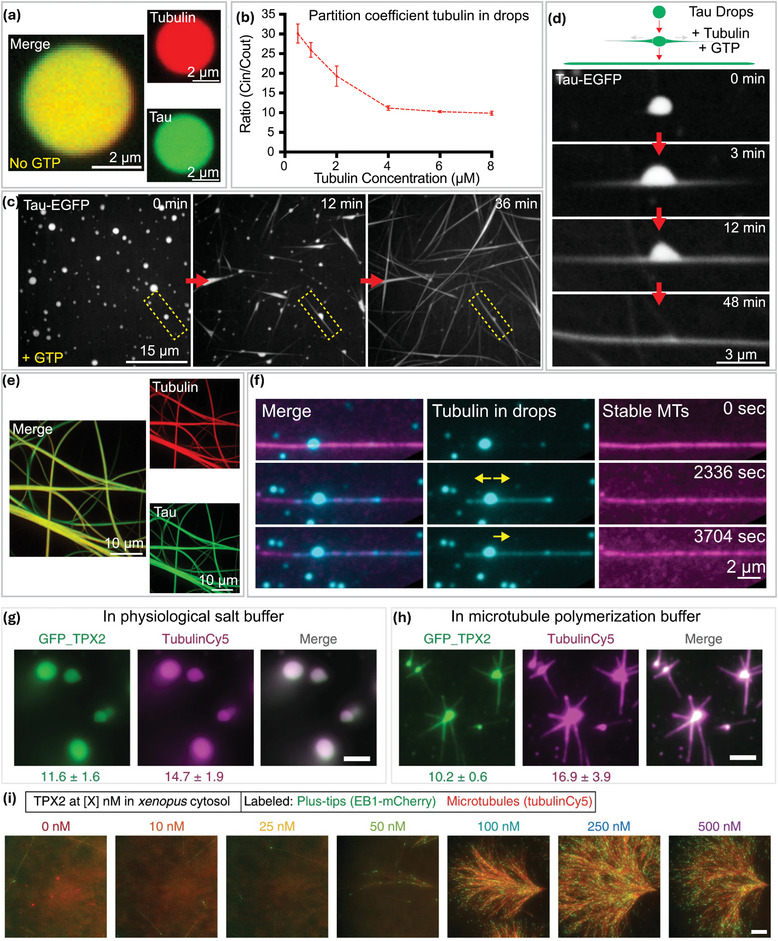
Controlled fabrication of microtubules. a) Tubulin can be effectively recruited in tau protein condensates. b) Tubulin partition coefficient in tau condensates. c) The polymerization of microtubule bundles is initiated from the tau droplets. d) The side view of single tau droplet deformation and polymerization of microtubule bundles. e) Colocalization of tau and tubulin on the nature microtubule bundles. f) Tubulin droplets can adhere to the existing microtubule and grow in the direction of the microtubule. g) Tubulin cocondensation with TPX2 protein molecules. Scale bar: 3 µm. h) When a polymerization buffer is applied, microtubules are developed from the TPX2‐tubulin condensates, expanding in multiple directions. Scale bar: 3 µm. i) Increasing TPX2 concentration leads to the nucleation and branching of microtubules. Scale bar: 10 µm. (a–f) Reproduced with permission.^[^ ^255^ ^]^ Copyright 2017, Elsevier. (g–i) Reproduced under the terms of the CC‐BY 4.0 license.^[^ ^256^ ^]^ Copyright 2020, Springer Nature.

Tubulin is the major component of microtubes. The self‐assembly of tubulin into microtubules follows a nucleation–polymerization process. It is found that some microtubule‐associated proteins can undergo LLPS to concentrate the tubulin and drive the nucleation of microtubules. For example, tau protein condensates can effectively recruit and concentrate tubulin molecules (**Figure** **9**a) with a partition coefficient ranging from 10‐ to 30‐fold (Figure 9b). It is demonstrated that by adding GTP into the solution, microtubule bundles nucleate from the droplets within 3 min and mature over 48 min (Figure 9c–e). Tubulin encapsulated in tau droplets can also grow along the existing microtubules (Figure 9f).^[^ ^255^ ^]^ Similarly, microtubule nucleation factor TPX2 can facilitate the microtubule growth initiated by the cocondensation with tubulin (Figure 9g).^[^ ^256^ ^]^ Unlike the bidirectional polymerization in the tau droplets (Figure 9d), tubulin in TPX2 condensates grows isotopically and forms a spike‐like structure (Figure 9h). Moreover, TPX2 condensates can coat the microtubules and form uniformly spaced droplets. These droplets can initiate the branching of microtubules (Figure 9i).^[^ ^256^, ^257^ ^]^ In conclusion, protein condensates play an important role in nucleation, formation, and branching of microtubules. They can eventually modulate the construction of microtubule networks, providing a broader functional scope for protein condensates in biomaterial engineering.

## 6 Conclusion and Outlooks

This review provides an overview of protein phase transitions, their underlying mechanisms in various biological processes, and their diverse roles in biofabrication. The precise design and arrangement of amino acid sequences enable proteins to assemble into liquid condensates and solid fibrils through LLPS and subsequent LSTs. These phase transitions can be influenced by PTMs, mutations, and various environmental factors. Effective modulation of protein phase behavior offers promising applications in drug discovery, delivery, and fabrication of multifunctional protein‐based liquid and solid materials. However, many fundamental aspects of protein phase behavior remain unclear, and current applications are still in their nascent stages.

Despite the ongoing efforts to reveal the molecular grammar of protein phase transitions, such as the sticker‐and‐spacer model,^[^ ^67^, ^258^ ^]^ LLPS and amyloid formation prediction tools,^[^ ^259^, ^260^, ^261^ ^]^ molecular dynamic simulations,^[^ ^262^, ^263^, ^264^ ^]^ and countless experimental studies, there is a significant gap remains in our understanding of how sequences contribute to phase transitions.^[^ ^66^, ^265^ ^]^ This hinders our ability to freely create protein‐based biomaterials with desired properties using de novo‐designed sequences. Further studies on the molecular mechanisms driving protein phase transitions will enhance our understanding and enable the effective use of these sequences in the development of bio‐inspired materials.

The widespread presence of nanoscale condensates, coupled with limitations in current probing tools, presents a challenge in studying the phase transitions of broader protostomes.^[^ ^21^, ^33^ ^]^ Specific analytical techniques also encounter other difficulties. For example, fluorophores may affect the protein phase behavior, and smFRET cannot provide atomic‐level details.^[^ ^266^ ^]^ While NMR and cryoEM can offer atomic‐level information, they struggle to capture the dynamicity and material properties of nanoscale condensates.^[^ ^266^ ^]^ We believe that with advancements in optical and analytical techniques, as well as other innovative single‐molecule probing approaches, a more detailed understanding of condensate LLPS and LSTs can be achieved in the future. These techniques will also help elucidate the role of various stimuli in the LSTs of protein condensates. Understanding the tolerance of the dissolution–reformation cycle and the slow aging of condensates under long‐term external stress is particularly crucial for studying condensate‐related diseases and applications such as stimuli‐responsive vesicles, switchable microreactors for biosynthesis, and artificial cells.

It is still not fully understood whether the LLPS of one protein can serve as a protective mechanism that delays its LST into irreversible amyloid fibrils. It would be intriguing to assess the extent to which LLPS can kinetically and thermodynamically trap protein molecules in their disordered states. Nevertheless, several studies have shown that the sequestration of aggregation‐prone proteins into condensates formed by other biomolecules can inhibit their irreversible aggregation.^[^ ^24^, ^25^ ^]^ This provides new insights into the prevention of protein aggregation‐related diseases.

Proteins from the hard and soft materials of living organisms offer valuable inspiration for the design of biomaterials. However, the phase behavior of a vast array of protein sources remains unexplored. For example, it is known that many food proteins can be engineered into amyloid fibrils and multiscale products upon denaturation and self‐assembly. It would be interesting to explore whether food proteins can undergo LLPS followed by LSTs, as well as their material properties and potential applications. For example, soy protein can be denatured and self‐assembled into amyloid fibrils.^[^ ^267^ ^]^ A later work discovered that glycinin from soy protein can undergo LLPS and form hollow droplets.^[^ ^268^ ^]^ Similarly, β‐lactoglobulin from milk can be engineered into amyloid‐fibril‐based bioproducts due to its enrichment of β‐strands.^[^ ^269^ ^]^ Recent works show that it can form liquid‐like condensates under crowded environments.^[^ ^270^, ^271^ ^]^ This suggests that food proteins can serve as an easily accessible resource for studying protein phase behavior. They also hold potential as a feedstock for developing protein‐based liquid and solid biomaterials, complementing recombinantly engineered proteins.

## Conflict of Interest

The authors declare no conflict of interest.
